# MicroRNA-Based Markers of Oral Tongue Squamous Cell Carcinoma and Buccal Squamous Cell Carcinoma: A Systems Biology Approach

**DOI:** 10.1155/2023/5512894

**Published:** 2023-04-24

**Authors:** Setareh Shojaei, Pouya Menbari, Shokoofeh Jamshidi, Amir Taherkhani

**Affiliations:** ^1^Department of Oral and Maxillofacial Pathology, School of Dentistry, Hamadan University of Medical Sciences, Hamadan, Iran; ^2^Department of Oral and Maxillofacial Pathology, School of Dentistry, Dental Research Center, Hamadan University of Medical Sciences, Hamadan, Iran; ^3^Research Center for Molecular Medicine, Hamadan University of Medical Sciences, Hamadan, Iran

## Abstract

**Objective:**

Oral tongue squamous cell carcinoma (OTSCC) and buccal squamous cell carcinoma (BSCC) are the first and second leading causes of oral cancer, respectively. OTSCC and BSCC are associated with poor prognosis in patients with oral cancer. Thus, we aimed to indicate signaling pathways, Gene Ontology terms, and prognostic markers mediating the malignant transformation of the normal oral tissue to OTSCC and BSCC.

**Methods:**

The dataset GSE168227 was downloaded and reanalyzed from the GEO database. Orthogonal partial least square (OPLS) analysis identified common differentially expressed miRNAs (DEMs) in OTSCC and BSCC compared to their adjacent normal mucosa. Next, validated targets of DEMs were identified using the TarBase web server. With the use of the STRING database, a protein interaction map (PIM) was created. Using the Cytoscape program, hub genes and clusters within the PIM were shown. Next, gene-set enrichment analysis was carried out using the g:Profiler tool. Using the GEPIA2 web tool, analyses of gene expression and survival analysis were also performed.

**Results:**

Two DEMs, including has-miR-136 and has-miR-377, were common in OTSCC and BSCC (*p* value <0.01; |Log2 FC| > 1). A total of 976 targets were indicated for common DEMs. PIM included 96 hubs, and the upregulation of EIF2S1, CAV1, RAN, ANXA5, CYCS, CFL1, MYC, HSP90AA1, PKM, and HSPA5 was significantly associated with a poor prognosis in the head and neck squamous cell carcinoma (HNSCC), while NTRK2, HNRNPH1, DDX17, and WDR82 overexpression was significantly linked to favorable prognosis in the patients with HNSCC. “Clathrin-mediated endocytosis” was considerably dysregulated in OTSCC and BSCC.

**Conclusion:**

The present study suggests that has-miR-136 and has-miR-377 are underexpressed in OTSCC and BSCC than in normal oral mucosa. Moreover, EIF2S1, CAV1, RAN, ANXA5, CYCS, CFL1, MYC, HSP90AA1, PKM, HSPA5, NTRK2, HNRNPH1, DDX17, and WDR82 demonstrated prognostic markers in HNSCC. These findings may benefit the prognosis and management of individuals with OTSCC/BSCC. However, additional experimental verification is required.

## 1. Introduction

Oral cancers (OCs) are a frequent type of malignancy in the head and neck region with a poor treatment outcome. Based on a previous report, the GLOBOCAN study estimated that 377,713 of the world's population would be affected by lip and oral cavity cancer in 2020, leading to 177,757 deaths [1]. The main risk factors for OCs are the use of tobacco and alcohol, exposure to UV radiation, and infection with the human papillomavirus (HPV) and Epstein–Barr virus (EBV) [2]. In addition, the aggressive nature of OC cells is strongly correlated with matrix metalloproteinase (MMP)-2, -9, and -13 [3]. Radical resection, radiotherapy, and chemotherapy are treatment approaches for OC patients. Among all the OC cases, 90% are classified as squamous cell carcinoma (SCC) [4]. Oral tongue SCC (OTSCC) is the most frequent subtype of OC and has the worst prognosis among all oral squamous cell carcinomas (OSCCs) [5–8]. Besides, buccal squamous cell carcinoma (BSCC) was reported as the second most common tumor in the oral cavity [9]. Despite advancements in therapeutic methods, the 5-year survival rate of OSCC patients has not improved efficiently. When the illness is discovered at stage IV, it drops from 80% to 30% when compared to primary OSCC [10]. By the appearance of clinical signs, up to 50% of OSCC patients are identified after they are already advanced [11]. In order to battle OSCC and increase patient survival rates, it is necessary to suggest more effective treatment options [12].

MicroRNAs (miRNAs) are noncoding and small RNAs (20–23 nucleotides) that regulate the transcription of their target genes; these upstream regulators bind to their complementary sequences at 3′ UTR of their mRNA targets, leading to mRNA degradation or translation inhibition [13–15]. New research revealed that miRNAs might drastically dysregulate the expression of genes linked to ER stress, necroptosis, pro- and antiapoptotic activity, and oncogenes [16, 17]. Therefore, miRNAs showed regulatory roles in critical biological procedures, including the cell cycle process, apoptosis and necroptosis, proliferation, and differentiation [18]. Consequently, miRNAs were defined as valuable biomarkers for the prognosis and diagnosis of patients with cancer, which have demonstrated satisfying results when assigned as therapeutic targets in cancer [12].

Accumulating evidence suggests that miRNAs play a critical role in the pathogenesis and prognosis of patients with head and neck squamous cell carcinoma (HNSCC). An earlier study by Dioguardi et al. [19] found a strong correlation between miR-31 overexpression and a poor prognosis in HNSCC patients. Additionally, the miR-196 family [20] and miR-155 [21] have been identified as potential predictive indicators of survival for HNSCC. Besides, Dioguardi et al. [22] showed the underexpression of miR-195 in patients with HNSCC and its excellent potential as an independent prognostic survival marker for HNSCC.

The present study hypothesized that miRNAs might participate in the tumorigenesis of OTSCC and BSCC. It was suggested that there are common differentially expressed miRNAs (DEMs) in the OTSCC and BSCC tissues compared to the normal oral mucosa, leading to abnormal expression of their target genes. These targets may be involved in several signaling pathways and biological processes (BPs), which may help elucidate the mechanisms underlying OSCC. Some other target genes might act as prognostic markers in patients with OSCC. Thus, utilizing the orthogonal partial least squares (OPLSs), common DEMs in OTSCC and BSCC in comparison to their respective healthy tissues were revealed. Then, validated targets of common DEMs were identified, a protein-protein interaction (PPI) network was constructed, hub genes within the network were illustrated, and the prognostic roles of hubs in HNSCC were evaluated using Kaplan–Meier curves. Furthermore, the enriched pathways and BP terms associated with the main clusters in the PPI network were identified.

The dataset GSE168227 from the Gene Expression Omnibus (GEO), available at https://www.ncbi.nlm.nih.gov/geo/, was considered for reanalyzing to examine the present hypothesis. The dataset was developed by Rajan et al. [23] to monitor the miRNA expression pattern in OTSCC, BSCC, and their adjacent oral mucosa to achieve a novel prognostic miRNA signature for OSCC. All tissue samples were from patients at the Regional Cancer Center's Head & Neck Clinic (Thiruvananthapuram, India). Patients with any chronic systemic condition or those who had already had cancer therapy were not allowed to participate in the trial. Control samples were attained from the patients undergoing oral and maxillofacial surgery for noncancer illnesses. All tissue specimens were immediately snap-frozen and stored in liquid nitrogen. The present study was confirmed by the Ethics Committee of Hamadan University of Medical Sciences, Hamadan, Iran (ethics no. IR.UMSHA.REC.1401.451).

## 2. Methods

### 2.1. Dataset Recovery and Statistical Analysis

The miRNA expression dataset GSE168227 [23], based on the platform GPL8227 (Agilent-019118 Human miRNA Microarray 2.0 G4470B), was downloaded as a TXT file from Gene Expression Omnibus (GEO), available at https://www.ncbi.nlm.nih.gov/geo [24]. The dataset contained 48 oral tissue samples, including OTSCC (*n* = 16), BSCC (*n* = 14), normal tongue samples (*n* = 8), and normal buccal specimens (*n* = 10). For feature selection, R programming (version 4.0.2) was used. DEMs in OTSCC and BSCC were found using OPLSs in comparison to the comparable normal tissues. A cut-off condition was set to *p* value <0.01 and |Log2 fold change (FC)| > 1 [25, 26]. Common DEMs involved in the malignant transformation of normal oral mucosa to OTSCC and BSCC were indicated. Furthermore, the Shiny apps web-based tool [27] showed the volcano plot of miRNAs in the dataset GSE168227.

### 2.2. PPI Network Analysis Based on Common DEM Targets

The experimentally validated targets of common DEMs were identified using the TarBase version 8 database, available at https://dianalab.e-ce.uth.gr/html/diana/web/index.php?r=tarbasev8/index [28]. Possible interactions among the targets with a combined score of 0.4 [29] were indicated using the STRING knowledge tool, available at http://string-db.org/ [30]. After removing disconnected nodes inside the protein interaction map (PIM) [31], the connected network was downloaded as a TSV format and subsequently imported into the Cytoscape 3.9.1 software [32], available at https://www.cytoscape.org to perform structural analysis. Hub nodes were awarded to proteins whose degree and betweenness were above two times the average and whose closeness exceeded the mean of the PPI network's nodes. In line with our earlier research [33], utilizing the MCODE plugin, the primary clusters within the PIM were also highlighted.

### 2.3. Gene Set Enrichment Analysis

Significant molecular pathways and Gene Ontology (GO) terms involved in the malignant transformation of normal oral mucosa to OTSCC and BSCC were unraveled using g:Profiler tool, available at https://biit.cs.ut.ee/gprofiler/gost [34]. The cut-off condition was set to the false discover rate (FDR) < 0.05 and the number of entities ≥10, following our previous study [35]. The results from two primary pathway sources, including Reactome [36] and the Kyoto Encyclopedia of Genes and Genomes (KEGGs) [37] databases, were considered for pathway enrichment analysis. Notably, common DEMs targets were taken into account for enrichment analyses of cellular component (CC) and molecular function (MF). At the same time, pathway and BP annotation enrichment analyses were given to the genes inside the clusters as input data [15].

### 2.4. Prognostic Role of Hub Genes

Regarding the critical role of hub genes in the pathogenesis of OTSCC and BSCC, the prognostic impact of hubs in the HNSCC was evaluated using the Gene Expression Profiling Interactive Analysis 2 (GEPIA2) database [38], available at http://gepia2.cancer-pku.cn/#index. By reanalyzing the raw RNA-seq expression data of malignant and healthy tissue samples from the TCGA and GTEx datasets, the GEPIA2 generates Kaplan–Meier curves. Prognostic indicators were defined as genes with the log-rank test and the hazard ratio (HR) *p* values <0.05. The prognostic role of the combination of genes was also evaluated [39].

### 2.5. Gene Expression Evaluation of Prognostic Markers

The mRNA expression patterns of prognostic genes were evaluated in HNSCC tissues (*n* = 519) and healthy samples (*n* = 44). It was performed by boxplot analysis provided by the GEPIA2 database [38].

## 3. Results

### 3.1. DEMs in OTSCC and BSCC

**Figure 1:**
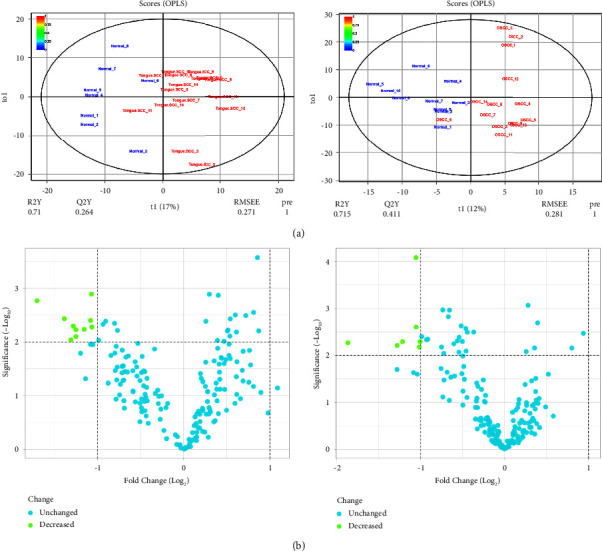
(a) Score plots in the predictive (*x*-axis) and orthogonal (*y*-axis) components of two datasets selected from GSE168227. (b) Volcano plots of miRNAs in OSCC compared to the normal oral tissues. Left and right images present results from OTSCC and BSCC tissues compared to their corresponding healthy samples, respectively. OTSCC, oral tongue squamous cell carcinoma; BSCC, buccal squamous cell carcinoma; OSCC, oral squamous cell carcinoma.

Two prediction models were created using OPLSs to find DEMs between the OTSCC and BSCC tissues in comparison to their healthy counterparts. Each model was built using 369 variables and 24 samples. At *p* value <0.01 and Log2 FC > 1, a total of 10 DEMs were indicated in OTSCC compared to the healthy oral mucosa (R2X = 0.494; R2Y = 0.71; and *Q*2 = 0.264). Besides, seven DEMs were identified in BSCC than in control samples (R2X = 0.526; R2Y = 0.715; and *Q*2 = 0.411) (Figure 1(a)). All DEMs in OTSCC and BSCC are listed in Table 1. Two DEMs, including has-miR-136 and has-miR-377, were common in two subtypes of OSCC. Volcano plots showed DEMs in the studies groups based on −Log 10 *p* value and Log 2 FC (Figure 1(b)).

### 3.2. Hub Genes, Modules, Pathways, and GO terms

**Figure 2:**
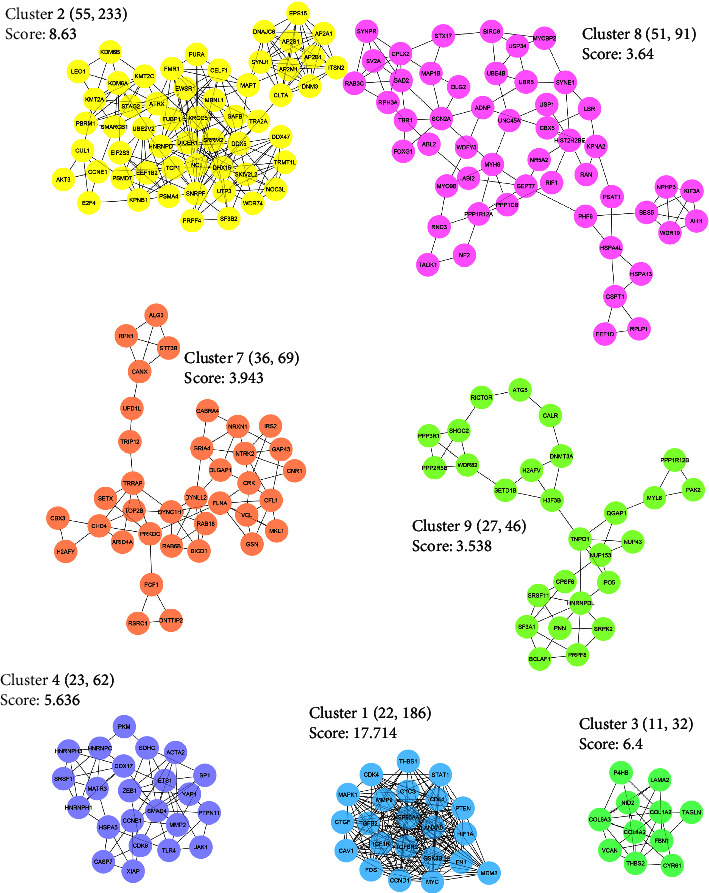
Seven clusters were identified by the MCODE plugin within the PIM associated with the pathogenesis of OTSCC and BSCC. The network was built based on validated targets of common DEMs in two subtypes of OSCC. MCODE, molecular complex detection; PIM, protein interaction map; OTSCC, oral tongue squamous cell carcinoma; BSCC, buccal squamous cell carcinoma; DEM, differentially expressed miRNA.

**Figure 3:**
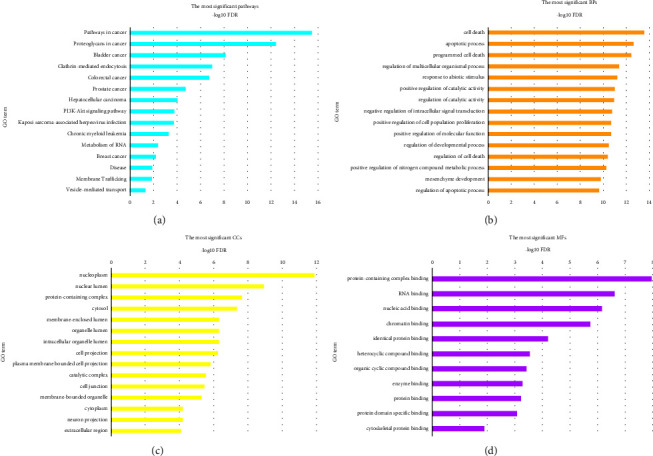
The most significant (a) pathways, (b) biological processes, (c) cellular components, and (d) molecular functions dysregulated in OTSCC and BSCC. The *x*-axis shows the pathway and GO term's names, while the *y*-axis demonstrates −Log10 of the false discovery rate. OTSCC, oral tongue squamous cell carcinoma; BSCC, buccal squamous cell carcinoma; GO, Gene Ontology.

The TarBase database detected 976 genes as experimentally validated targets for common DEMs. The list of targets was used as input data in the STRING database to construct a PPI network. Single nodes were removed from the PIM, and subsequently, a connected network including 932 proteins and 6682 interactions was imported into Cytoscape. The average degree, betweenness, and closeness value was calculated as 14.33, 0.0023, and 0.33, respectively. Ninety-six nodes were then identified as hub genes linked to the pathogenesis of OTSCC and BSCC. (Table 2). Further structural analysis was performed using the MCODE plugin. Seven substantial clusters (cluster No. 1, cluster No. 2, cluster No. 3, cluster No. 4, cluster No. 7, cluster No. 8, and cluster No. 9) were found to be involved in the pathways and BPs linked to the pathogenesis of OTSCC and BSCC. Cluster No. 1 demonstrated the most MCODE score (MCODE score = 17.714), and cluster No. 2 included the most number of genes (*n* = 55) (Figure 2). The most important pathways implicated in the carcinogenesis of OTSCC and BSCC also included “pathway in cancer” (KEGG:05200), “proteoglycan in cancer” (KEGG:05205), “bladder cancer” (KEGG:05219), and “clathrin-mediated endocytosis” (REAC: R-HSA-8856828). The two most essential BPs promoting malignant transformation in the tongue and buccal area were “cell death” (GO:0008219) and “apoptotic process” (GO:0006915). Moreover, “neoplasm” (GO:0005654) and “nuclear lumen” (GO:0031981) CCs were considerably affected in OTSCC and BSCC. Moreover, “Protein-containing complex binding” (GO:0044877) and “RNA binding” (GO:0003723) were the most enriched terms in the category of MFs.  Figure 3 demonstrates the most significant pathways and GO terms dysregulated in the pathogenesis of OTSCC and BSCC.

### 3.3. Prognostic Markers

**Figure 4:**
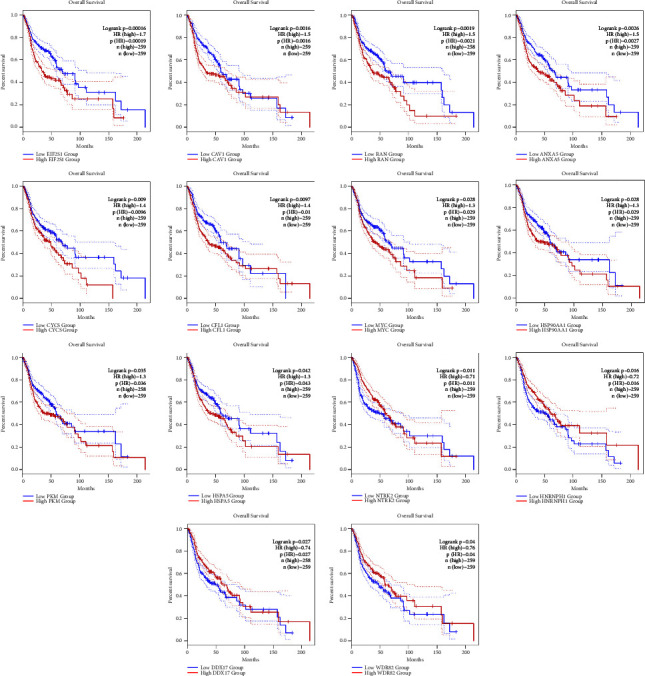
Survival analysis of EIF2S1, CAV1, RAN, ANXA5, CYCS, CFL1, MYC, HSP90AA1, PKM, HSPA5, NTRK2, HNRNPH1, DDX17, and WDR82 in patients with HNSCC. Blue and red lines show under- and overexpressed markers, respectively. The *y*-axis and *x*-axis demonstrate the probability of survival and survival months of patients with HNSCC, respectively. The dotted lines show a 95% confidence interval. HNSCC, head and neck squamous cell carcinoma.

**Figure 5:**
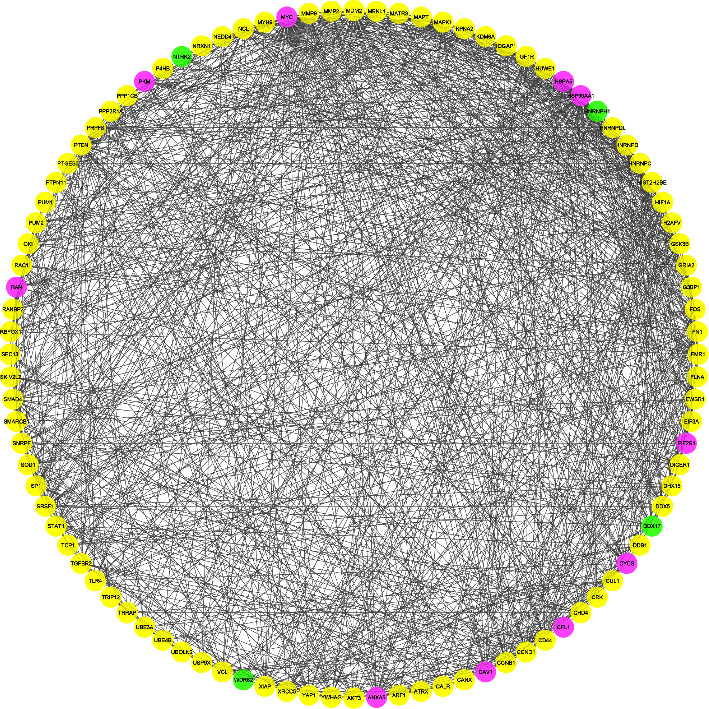
A connected PPI network based on the hub genes. Violet and green circles represent negative and positive markers in HNSCC, respectively. PPI, protein-protein interaction network; HNSCC, head and neck squamous cell carcinoma.

Kaplan–Meier curves showed that the overexpression of EIF2S1, CAV1, RAN, ANXA5, CYCS, CFL1, MYC, HSP90AA1, PKM, and HSPA5 was significantly associated with a dismal prognosis in the patients with HNSCC. EIF2S1 showed the most negative marker with the criteria of HR = 1.7, log-rank test *p* value = 0.00016, and HR *p* value = 0.00019. Additionally, an improved prognosis in HNSCC was associated with the overexpression of NTRK2, HNRNPH1, DDX17, and WDR82. With an HR, log-rank *p* value, and HR *p* value of 0.71, 0.011, and 0.011, respectively, NTRK2 was the most positive marker. Therefore, these markers might be assigned as drug targets in patients with OTSCC and BSCC.  Figure 4 presents the survival analysis of prognostic markers.  Figure 5 shows interactions between hub genes. The combination of EIF2S1, CAV1, RAN, and ANXA5 showed a considerable negative signature with the criteria of HR = 1.6, log-rank *p* value = 0.00028, and HR *p* value = 0.00032 (Table 3).

### 3.4. Gene Expression of Markers

**Figure 6:**
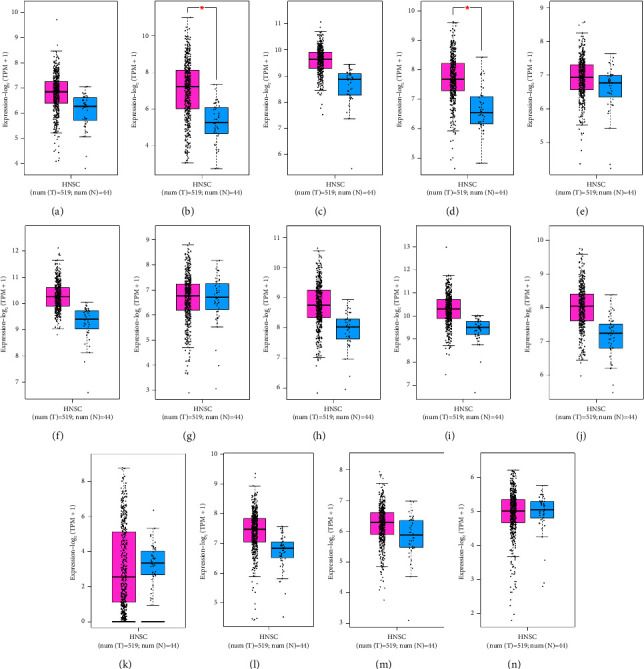
Gene expression patterns of prognostic markers in HNSCC including (a) EIF2S1, (b) CAV1, (c) RAN, (d) ANXA5, (e) CYCS, (f) CFL1, (g) MYC, (h) HSP90AA1, (i) PKM, (j) HSPA5, (k) NTRK2, (l) HNRNPH1, (m) DDX17, and (n) WDR82. Box plots are based on 519 HNSCC tissues (pink color) and 44 healthy oral tissues (blue color). HNSCC, head and neck squamous cell carcinoma.

Based on boxplot analysis, the mRNA levels of EIF2S1, CAV1, RAN, SELE, ANXA5, CYCS, CFL1, HSP90AA1, PKM, HSPA5, HNRNPH1, and DDX17 exhibited a significant overexpression in HNSCC than in healthy controls. NTRK2 demonstrated a considerable underexpression in HNSCC than in normal oral mucosa. The MYC and WDR82 expressions were insignificant (Figure 6).

## 4. Discussion

The incidence of OTSCC and BSCC is considered the first and second highest among all oral cancers, respectively [40, 41]. MMP-13 inhibitors [42] may be effective treatments for improving survival rates in patients with OTSCC and BSCC because of the substantial role MMP-13 plays in the invasion of OC cells. However, unraveling the most critical genes, molecular pathways, and GO annotations mediating the malignant transformation of normal oral mucosa to OTSCC and BSCC might be helpful in treating OSCC. Gene set enrichment analysis showed that the “Clathrin-mediated endocytosis” pathway (REAC: R-HSA-8856828) significantly mediates the malignant transformation of normal oral mucosa to OTSCC and BSCC. “Clathrin-mediated endocytosis” (CME) is an endocytic process that regulates the expression of plasma membrane receptors and influences the pathways that lead to those receptors' downstream signals [43–45]. Xiao et al. [46] showed that ERK1/2 phosphorylates the FCH/F-BAR and SH3 domain-containing protein (FCHSD2), leading to enhanced clathrin-coated pit (CCP) initiation and CME, resulting in decreased cell-surface EGFR expression and reduced proliferation and migration of nonsmall cell lung cancer cells.

The present study illustrated that hsa-miR-377 and hsa-miR-136 are commonly downregulated in OTSCC and BSCC compared to the normal oral mucosa. Sun et al. [47] reported a significant downregulation of miR-377-3p in nonsmall cell lung cancer (NSCLC) tissues compared to their corresponding healthy lung specimens. Besides, Sun et al. [47] demonstrated a remarkable negative correlation between the expression of oncogene E2F3 mRNA and miR-377-3p in lung tissues using Pearson correlation analysis (*r*2 = 0.3614, *p* < 0.0001), suggesting the tumor suppressive role of miR-377-3P by downregulating the E2F3. The authors noted that the elevation of the long noncoding RNA (lncRNA) NEAT1 prevented the increased cell death caused by miR-377-3p. NEAT1 overexpression was linked to carcinogenesis and cancer development, and NEAT1 has been described as an oncogenic gene in several malignancies [48, 49]. Evidence suggests that estrogen receptor-*α*36 (ER*α*36) plays a significant role in the tumorigenesis of luminal subtypes of breast cancer. In this regard, the enhanced expression of ER*α*36 is associated with disease development and drug resistance [50–52]. Thiebaut et al. [53] showed a negative correlation between the expression of the miR-136-5p and ER*α*36 in breast cancer cells. The miR136-5p mimic transfection in MCF-7 cells diminished the ER*α*36 expression.

OSCC is the most common HNSCC [54], counting for 95% of all HNSCC cases [55]. Furthermore, the GEPIA2 (or GEPIA) database for HNSCC is commonly used to explore the prognostic effect of genes in OSCC and/or to evaluate the mRNA expression levels of genes in OSCC compared to healthy controls. Using immunohistochemical analysis, Sun et al. [56] compared the protein expression levels of AKT1 and PLK1 in OSCC tissues to normal oral specimens. Subsequently, Sun et al. [56] used the GEPIA web server to validate their experimental results; this included evaluating the mRNA expression levels of AKT1 and PLK1 in HNSCC than in healthy tissues and analyzing the prognostic effect of the genes in patients with HNSCC. Fang et al. [57] identified several genes with a correlation score >0.8 in a PPI network associated with the pathogenesis of OSCC. Next, the authors used the GEPIA tool to evaluate the prognostic role of the genes in HNSCC. Furthermore, Dai et al. [58] performed a weighted gene comethylation network analysis (WGCNA) to identify hub modules and CpG sites correlated with OSCC. Then, Dai et al. [58] used the GEPIA for HNSCC to conduct a Kaplan–Meier survival analysis to investigate the possible predictive significance of the numerous hub CpG site-associated genes. The GEPIA2 for HNSCC was used in the present study to investigate the potential prognostic impact of the hub genes in OTSCC and BSCC. Gene expressions of the prognostic markers were also evaluated using GEPIA2 for HNSCC.

Based on the targets of hsa-miR-377 and hsa-miR-136, a PPI network of 932 genes and 6682 edges was created here. EIF2S1, CAV1, RAN, ANXA5, CYCS, CFL1, MYC, HSP90AA1, PKM, and HSPA5 overexpression was substantially related to a poor prognosis in patients with HNSCC, and 96 hub genes showed a striking centrality within the PPI network. Besides, the overexpression of NTRK2, HNRNPH1, DDX17, and WDR82 was linked to a favorable prognosis in HNSCC patients (the log-rank test and HR *p* values <0.05).

Eukaryotic translation initiation factors were introduced as novel drug targets in many cancers [59]. Eukaryotic translation initiation factor 2 subunit 1 (EIF2S1 [EIF2A]) mediates the binding of Met-tRNAi to the 40S/mRNA complex [60]. Several reports have linked the missregulation of eukaryotic translation initiation factors to abnormal cell growth and tumor development [61–63]. Additionally, angiogenesis and metastasis in cancer cells are linked to the PERK/eIF2a signaling pathway [64, 65]. Intestinal-type adenocarcinoma is rare cancer affecting the nasal cavity and paranasal sinuses. Schatz et al. [66] found that it significantly overexpressed EIF2S1, EIF5A, and EIF6 when compared to healthy tissue samples. Recently, Li et al. [67] linked the UTP14A overexpression in oesophageal squamous cell carcinoma (ESCC) cells to the upregulation of PERK/eIF2a signaling pathway, leading to the cell cycle process and migration of ESCC cells. The overexpression of EIF2S1 was confirmed at the mRNA level in HNSCC compared with healthy tissues.

Lu et al. [68] performed a study to elucidate the role of caveolin-1 (CAV-1) and ferroptosis on HNSCC development. The research findings by Lu et al. [68] showed that CAV-1 was markedly overexpressed in HNSCC compared to healthy tissues. The ferroptosis process was significantly inhibited by CAV-1, which increased cell proliferation, invasion, and metastasis. CAV-1 was significantly associated with a dismal prognosis in HNSCC patients as well. Therefore, CAV-1 was assigned as a potential target in HNSCC.

The present study had several limitations. The dataset GSE168227 only included patients from the Head and Neck Clinic of Regional Cancer Centre (Thiruvananthapuram, India). As a result, patients from other nations could not completely benefit from the current results. Additionally, the sample size was small since the GSE168227 only included 48 oral tissue samples. A set of miRNAs selected in the dataset GSE168227 was based on the GPL8227 platform, which may not symbolize all the miRNAs.

## 5. Conclusion

The present study suggests has-miR-136 and has-miR-377 as common DEMs in OTSCC and BSCC compared to the normal oral mucosa (*p* value <0.01; |Log2 FC| > 1). A total of 976 genes were identified as validated targets of common DEMs. Ninety-six genes showed salient centrality within the PIM mediating the tumorigenesis of OTSCC and BSCC, in which EIF2S1, CAV1, RAN, ANXA5, CYCS, CFL1, MYC, HSP90AA1, PKM, and HSPA5 were significantly linked to a poor prognosis in HNSCC. In addition, individuals with HNSCC had a better prognosis when they had NTRK2, HNRNPH1, DDX17, and WDR82 overexpression. “Clathrin-mediated endocytosis” was significantly enriched in OTSCC and BSCC. Our findings might improve the prognosis of patients with OTSCC/BSCC, leading to more effective therapeutic strategies. However, in vitro and in vivo confirmations are needed in the future.

## Figures and Tables

**Table 1:** Differentially expressed miRNAs in OTSCC and BSCC compared with the normal oral mucosa identified by OPLS.

*A, DEMs in OTSCC*			

*MicroRNA ID*	*p value*	*FC*	*Log2 FC*

hsa-miR-136	0.002	0.307	−1.702
hsa-miR-30e	0.004	0.383	−1.386
hsa-miR-95	0.009	0.403	−1.311
hsa-miR-338-3p	0.005	0.411	−1.283
hsa-miR-376a	0.006	0.420	−1.251
hsa-miR-144	0.008	0.420	−1.251
hsa-miR-1	0.006	0.449	−1.155
hsa-miR-337-5p	0.004	0.473	−1.081
hsa-miR-133a	0.001	0.476	−1.070
hsa-miR-377	0.005	0.478	−1.066

*B, DEMs in BSCC*			

*MicroRNA ID*	*p value*	*FC*	*Lof2 FC*

hsa-miR-136	0.005	0.274	−1.868
hsa-miR-299-5p	0.006	0.412	−1.280
hsa-miR-144	0.005	0.430	−1.217
hsa-miR-139-5p	0.000	0.481	−1.055
hsa-miR-30a	0.002	0.482	−1.053
hsa-miR-377	0.007	0.494	−1.016
hsa-miR-204	0.005	0.498	−1.006

OTSCC, oral tongue squamous cell carcinoma; BSCC, buccal squamous cell carcinoma; DEM, differentially expressed miRNA; FC, fold change.

**Table 2:** A total of 96 nodes demonstrated high centrality values in the PPI network associated with OTSCC and BSCC and considered hub genes.

Gene symbols	Degrees	Betweenness	Closeness
MYC	149	0.076	0.483
HSP90AA1	130	0.060	0.472
HSPA5	78	0.033	0.435
PTEN	106	0.032	0.449
FN1	88	0.026	0.425
MDM2	76	0.026	0.432
MAPT	65	0.025	0.427
MAPK1	76	0.023	0.431
SMAD4	73	0.018	0.418
RAC1	62	0.018	0.410
GSK3B	72	0.017	0.427
CCNB1	59	0.017	0.416
CUL1	48	0.017	0.398
CCND1	84	0.016	0.428
ARF1	37	0.016	0.382
FOS	62	0.016	0.416
HIF1A	75	0.016	0.428
HIST2H2BE	67	0.016	0.414
HNRNPC	75	0.015	0.417
CANX	44	0.015	0.396
NCL	62	0.014	0.414
CAV1	57	0.014	0.414
PPP1CB	49	0.014	0.407
G3BP1	55	0.014	0.409
GRIA2	43	0.013	0.390
HUWE1	46	0.013	0.401
FMR1	49	0.013	0.409
DICER1	51	0.012	0.402
PUM2	41	0.012	0.385
STAT1	51	0.012	0.406
IGF1R	54	0.012	0.419
NRXN1	39	0.011	0.379
NTRK2	38	0.011	0.389
SRSF1	68	0.011	0.407
RANBP2	43	0.011	0.395
UBE3A	41	0.011	0.403
DDX5	52	0.011	0.400
SEC13	41	0.011	0.384
UBE4B	30	0.011	0.375
PTPN11	46	0.010	0.399
P4HB	41	0.010	0.389
IQGAP1	48	0.010	0.406
TRIP12	36	0.010	0.384
HNRNPH1	56	0.010	0.393
YWHAG	34	0.010	0.393
CYCS	49	0.009	0.411
HNRNPD	51	0.009	0.398
YAP1	44	0.009	0.395
DHX15	56	0.009	0.387
VCL	41	0.009	0.384
QKI	33	0.009	0.387
DDB1	40	0.009	0.392
CRK	35	0.009	0.386
RAN	43	0.009	0.387
XRCC5	44	0.008	0.396
SNRPF	45	0.008	0.371
EIF2S1	43	0.008	0.400
CD44	57	0.008	0.402
TLR4	48	0.008	0.397
CHD4	56	0.008	0.391
CFL1	39	0.008	0.392
KDM6A	42	0.008	0.391
H2AFV	42	0.008	0.379
CALR	40	0.008	0.399
PPP2R1A	35	0.008	0.397
USP9X	36	0.007	0.390
XIAP	42	0.007	0.404
DDX17	45	0.007	0.394
MATR3	46	0.007	0.393
SP1	36	0.007	0.391
SOD1	36	0.007	0.399
HNRNPDL	48	0.007	0.392
PTGES3	37	0.007	0.390
PRPF8	46	0.007	0.380
SKIV2L2	43	0.007	0.364
PUM1	35	0.007	0.370
TRRAP	36	0.007	0.383
EWSR1	43	0.007	0.398
NEDD4	30	0.006	0.384
FLNA	44	0.006	0.390
UBQLN2	38	0.006	0.394
PKM	35	0.006	0.396
WDR82	35	0.006	0.374
RBFOX1	29	0.006	0.364
MYH9	31	0.006	0.379
KPNA2	35	0.005	0.391
AKT3	29	0.005	0.383
ATRX	41	0.005	0.384
TGFBR2	38	0.005	0.387
MMP2	47	0.005	0.385
MBNL1	30	0.005	0.368
TCP1	33	0.005	0.381
EIF3A	31	0.005	0.364
ANXA5	50	0.005	0.412
MMP9	52	0.005	0.400
SMARCB1	41	0.005	0.389

PPI, protein-protein interaction; OTSCC, oral tongue squamous cell carcinoma; BSCC, buccal squamous cell carcinoma.

**Table 3:** A total of 14 hub genes in the PIM associated with the etiology of OTSCC and BSCC were revealed to be prognostic markers in HNSCC.

*A, single gene*			

*Gene symbols (labels)*	*HR (high)*	*P (log-rank test)*	*P (HR)*

EIF2S1 (a)	1.7	0.00016	0.00019
CAV1 (b)	1.5	0.0016	0.0016
RAN (c)	1.5	0.0019	0.0021
ANXA5 (d)	1.5	0.0026	0.0027
CYCS (e)	1.4	0.009	0.0096
CFL1 (f)	1.4	0.0097	0.01
MYC (g)	1.3	0.028	0.029
HSP90AA1 (h)	1.3	0.028	0.029
PKM (i)	1.3	0.035	0.036
HSPA5 (j)	1.3	0.042	0.043
NTRK2 (k)	0.71	0.011	0.011
HNRNPH1 (l)	0.72	0.016	0.016
DDX17 (m)	0.74	0.027	0.027
WDR82 (n)	0.76	0.04	0.04

*B, combination of genes*			

*Prognostic signature*	*HR (high)*	*P (log-rank test)*	*P (HR)*

a and b	1.4	0.01	0.01
a to c	1.4	0.013	0.014
a to d	1.6	0.00028	0.00032
a to e	1.5	0.0029	0.0031
a to f	1.6	0.0012	0.0013
a to g	1.5	0.0041	0.0044
a to h	1.6	0.0004	0.00045
a to i	1.6	0.00084	0.00092
a to j	1.6	0.00046	0.00052

PIM, protein interaction map; OTSCC, oral tongue squamous cell carcinoma; BSCC, buccal squamous cell carcinoma; HNSCC, head and neck squamous cell carcinoma.

## Data Availability

The datasets used and/or analyzed during the current study are available from the corresponding author upon reasonable request.
